# HetEnc: a deep learning predictive model for multi-type biological dataset

**DOI:** 10.1186/s12864-019-5997-2

**Published:** 2019-08-08

**Authors:** Leihong Wu, Xiangwen Liu, Joshua Xu

**Affiliations:** 1 Division of Bioinformatics and Biostatistics, National Center for Toxicological Research, U.S. Food and Drug Administration, 3900 NCTR Rd, Jefferson, AR 72079 USA; 2 Department of Information Science, University of Arkansas at Little Rock, 2801 S. University Ave., Little Rock, AR 72204 USA

## Abstract

**Background:**

Researchers today are generating unprecedented amounts of biological data. One trend in current biological research is integrated analysis with multi-platform data. Effective integration of multi-platform data into the solution of a single or multi-task classification problem; however, is critical and challenging. In this study, we proposed HetEnc, a novel deep learning-based approach, for information domain separation.

**Results:**

HetEnc includes both an unsupervised feature representation module and a supervised neural network module to handle multi-platform gene expression datasets. It first constructs three different encoding networks to represent the original gene expression data using high-level abstracted features. A six-layer fully-connected feed-forward neural network is then trained using these abstracted features for each targeted endpoint. We applied HetEnc to the SEQC neuroblastoma dataset to demonstrate that it outperforms other machine learning approaches. Although we used multi-platform data in feature abstraction and model training, HetEnc does not need multi-platform data for prediction, enabling a broader application of the trained model by reducing the cost of gene expression profiling for new samples to a single platform. Thus, HetEnc provides a new solution to integrated gene expression analysis, accelerating modern biological research.

## Background

The use of integrated analysis with multi-platform gene expression data in current biological research is increasing [1–4]. In general, “multi-platform” refers to data from multiple technologies or from different sites/tissues/organs, which usually have close linkages or relationships between data platforms. For example, the Sequencing Quality Control (SEQC) project [5, 6] studied a large neuroblastoma cohort with both Microarray (Agilent) and RNA-seq (Illumina Hi-seq) datasets. Genotype-Tissue Expression (GTEx), provided 1641 samples, covering multiple tissue or body sites, from 175 individuals [2].

These well-established and publicly available resources have provided a huge opportunity for developing integrative analysis approaches to gain more comprehensive insights. A particular interest is to build predictive models that integrate multi-platform data for enhanced performance. However, handling multi-platform data effectively is quite challenging. The difficulties mostly come from the inability to utilize the complicated, close linkages among features from different platforms efficiently. Several reviews have been conducted in integrative models [7, 8]. Popular integrated analysis includes horizontal (or simultaneous) and vertical (or sequential) data integration [9], However, both of which assumes every sample, including the testing data has the data accessibility of all platforms.

Deep learning, one of the most promising methods in current machine learning, has been implemented in a variety of research fields, including object recognition, keyword triggering, language translation, and others [10]. It has been applied in such biological study areas as variant calling [11, 12], protein-binding prediction [13], predicting variant chromatin effects [14], and biomedical imaging [15–17]. Note that most of these biological applications were applied to spatial/temporal/sequential data, for which many deep learning approaches have been developed in other research fields. For example, convolutional neural network (CNN) [18, 19] has been widely applied to image analysis, including bioimaging; a recurrent neural network (RNN) [20, 21] was designed to, and was capable of, handling sequential data such as text documents, soundtracks, and DNA sequences. However, to our knowledge, few deep learning approaches were developed for tabular datasets, such as those for gene expression, which is one of the most common data types in current biological research [22]. Since features in tabular data didn’t have temporal order, CNN and RNN frameworks are usually not applicable, unless external linkage information (such as pathway, GO function, genome location, etc.) was further added onto the dataset. In other words, the linkage information between features was collected from external resource but not directly extracted from the dataset itself, therefore may bring more restrictions to the following data analysis. For example, a deep neural network constructed based on pathway information may not well handle genes which does not have much pathway information.

Here we propose *HetEnc*, a deep learning approach for integrated gene expression analysis, which integrates different platforms of genomics features on the same cohort of subjects. HetEnc is designed as two sequential modules. In the first module of feature representation, it utilizes the multi-platform information in an unsupervised fashion to generate a high-level abstracted feature set, also known as intermediate features. In the second module of predictive modeling, a deep feed-forward neural network is constructed using the intermediate features as input, to train the model for each targeted endpoint.

## Methods

### SEQC neuroblastoma dataset

The SEQC neuroblastoma dataset includes 498 neuroblastoma patients’ gene expression profiles measured both by Microarray and RNA-seq. The training dataset consisted of 249 samples, and the other 249 samples were in the validation/test dataset. We used the sample distribution as defined in SEQC project [4–6].

The expression profile for both Microarray and RNS-seq analyses currently are publicly available in the National Center for Biotechnology Information (NCBI) GEO database. The Microarray data (GEO accession: GSE49710) was generated using customized 4x44k oligonucleotide microarrays (Agilent Technologies) and extracted via Agilent’s Feature Extraction software (Ver. 9.5.1). The RNA-seq sequencing data (GEO accession: GSE62564) was performed on the Hi-Seq 2000 platform (Illumina). Detailed sample preparation and data pre-processing has been described elsewhere [4].

We investigated three clinical endpoints from the neuroblastoma dataset, including favorable prognosis (FAV), overall survival (OS_All), and high-risk patient survival (OS_HR). FAV is a binary label for patients belonging to a favorable subgroup that is event-free (i.e., no progression, relapse, death) without chemotherapy for at least 1000 days, or those belonging to an unfavorable subgroup that died from disease despite chemotherapy. OS is the occurrence of death from disease, and high risk (HR) only includes patients belonging to a high-risk subgroup (with stage 4 disease > 18 months at diagnosis or with MYCN-amplified tumors at any age and stage). Based on previous experience, these three endpoints have different levels of predictability: FAV is usually easy to predict and has a high predictive performance in all modeling algorithms, whereas OS_HR is very difficult to predict no matter which modeling algorithm is applied. As reported, the predictive difficulty of OS_All falls between FAV and OS_HR. Since not all clinical endpoints were available, the FAV and OS_HR study did not include all 498 samples. A detailed description of these three endpoints and their previous predicting performance are summarized in Table 1**.**

**Table 1:** Summary of Neuroblastoma Endpoints

Endpoint	FAV	OS_All	OS_HR
Full description	Neuroblastoma Favorable Prognosis	Overall Survival	Survival in High Risk patients
Sample size (Train/Test)	136/136	249/249	86/90
Train set prevalence	45/91 (0.669)	51/198 (0.795)	43/43 (0.500)
Test set prevalence	46/90 (0.662)	54/195 (0.783)	49/41 (0.544)
Predicting difficulty (Zhang, et al., 2015)	Easy	Medium	Hard

### Data pre-processing

All SEQC neuroblastoma datasets were already pre-processed when downloaded from the GEO database. While there were several available data pre-processing pipelines for RNA-seq data, we chose the dataset pre-processed by Su, et al. (2014), to focus on the subset of 10,042 genes that were mapped one-to-one between Microarray and RNA-seq. Therefore, the final data matrix of train and test datasets were (249, 10,042) and (249, 10,042), for both Microarray and RNA-seq platforms, meaning that the notation of (249, 10,042) is 249 samples with 10,042 (gene expression) features for each sample.

### Feature representation with unsupervised learning

Unsupervised Learning is a topic of interest in today’s deep learning community. One typical unsupervised learning algorithm is autoencoder (AE), which is designed to compress high-dimension data into low-dimension features. A typical AE is composed of two connected networks: an encoding network and a decoding network. The encoding network tries to compress the input data into low-dimensional features, which made up the bottleneck (layer); the decoding network, in reverse, tries to reconstruct the original input data from the low-dimensional features. In a combination of the encoding and decoding networks, the AE is much like a regular Multilayer Perceptron (MLP), where the major difference is that the input and expected output of this MLP are the same. In other words, the learning process of this AE tries to reconstruct the input data with minimal information loss.

**Fig. 1:**
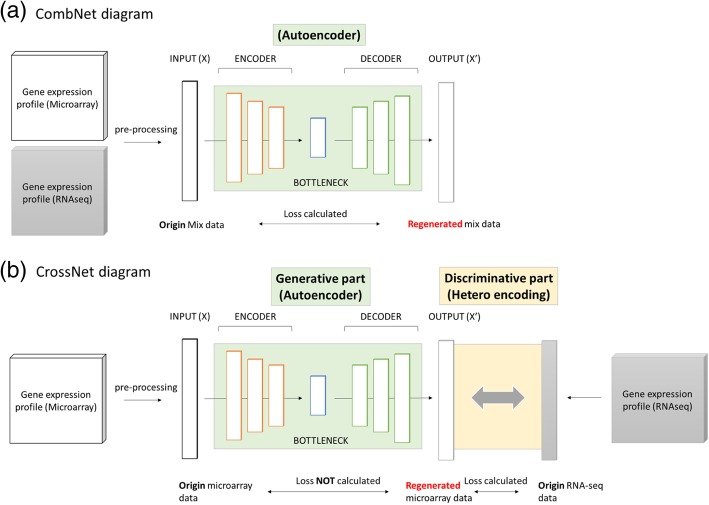
(**a**) Diagram of CombNet. Microarray and RNA-seq data were mixed before entering the autoencoder. Same feature spaces were defined in both platforms (**b**) Diagram of CrossNet. The first part (generative part) is an autoencoder, where an encoder and decoder are combined to regenerate microarray gene expression profile. The second part (discriminative part) is then introduced to reduce the difference between regenerated microarray data (i.e., the output of generative part) and origin RNA-seq data. In current version, we do not build another discriminative model but use the crossentropy to simplify the process

A novel aspect of this study is that we not only used the regular AE for one platform; we also designed other two kind of representation networks. The first network is named as CombNet, which first combined two different gene expression data together, treated them as the same type of data that could be represented by single autoencoder (Fig. 1a). Particularly, we used the overlapped 10,042 genes as the feature space in both platforms. The second network is named as CrossNet, where the input and expected output are not identical; in such a case the network tries to learn the representations that could be bridge the conversion of one platform to another (Fig. 1b). In CrossNet, there are two parts of modules, the first part, or the generative part is an autoencoder that try to regenerate data from one platform (such as microarray) with updated weights from the second part. The second part compared the regenerated microarray data and the origin RNA-seq data (i.e. second platform), in order to reduce their differences. The final goal of the CrossNet model is to find out the bottleneck layer of the generative part that minimize the loss in discriminative part, somehow similar to the Generative Adversarial Networks (GANs).

### Predictive model based on deep neural network

In the predictive modeling step, we applied a fully-connected neural network with feed-forward architecture. A fully-connected neural network is an artificial neural network format with at least three layers: one input layer, one output layer and one hidden layer. Fully-connected means linkages exist among nodes between two adjacent layers; however, there are no linkages between nodes in the same layer. Usually, there is more than one hidden layer in a deep neural network architecture, and HetEnc used four of these in the modeling step. Feed-forward means the network did not have a connection forming a cycle, unlike Boltzmann machine and recurrent neural networks.

The linkages between nodes in two adjacent layers could be either linear (i.e., forms z_1_ = Wa_0_ + b) or non-linear (i.e., *rectified linear unit*, *logistic function*, etc.). Usually, for a non-linear function, an activation function can be added to a linear basis, making the whole function non-linear (i.e., a_1_ = *f*(z_1_), where *f* is the non-linear activation function and z_1_ is the output of a linear function).

When training the neural network model, we used backpropagation to update the network weight between the training epochs. We used mini-batch (x = 32) gradient descent in backpropagation and the loss function for both datasets was *categorized cross-entropy*. The activation function of the output layer was *Softmax*.

The source code for the whole HetEnc is available at: https://github.com/seldas/HetEnc_Code.

### Other machine learning algorithms

In this study, we compared HetEnc models to three kinds of machine learning algorithms.

#### Previously established predictive models

In our previous study, three different types of predictive models, as K-Nearest Neighbors (KNN), Support Vector Machine (SVM) and Nearest shrunken centroids (NSC), were constructed by using the exact same pre-processed dataset. In general, gene features were pre-filtered by their *p*-value (< 0.05) and log2 fold-change (> 1.5). Parameter K in KNN ranged in (1, 3, 5, 7, 9); kernel used in SVM is ‘rbf’; and the other parameters are set as default. In training process, each model was trained based on randomly selected 70% of training data and its performance was evaluated on the remaining 30% of training data. The training process repeated 500 times to retrieve an overall cross-validation modeling performance. The model was then tested on the other 249 testing samples. In this study, we only used the model testing performance for comparison, which was averaged among 500 models. This part of the experiment was performed in R, with packages of ‘*class’* for KNN, ‘*pamr’* for NSC, and ‘*kernlab’* for SVM. More detailed description of these three models are published elsewhere.

#### Other popular tree-based predictive models

Besides KNN, SVM and NSC, we also compared HetEcn to two more commonly-used machine learning models, as Random Forest and XGBoost, using the same processed datasets. For Random Forest, we tuned the number of trees from 10 to 200, and observed a saturated performance when trees = 100. For XGBoost, tree-based models were selected as default. The other parameters are used as default. The training process of Random Forest was repeated 100 times, for each time the whole training dataset was used to train the model; and the model was then evaluated on the other 249 testing samples. Similarly, we only used the model testing performance for comparison, which was the average AUC among 100 repeats. Since XGBoost performance will not change when different random seed was set, we only ran XGBoost once. This part of the experiment was performed in Python, with modules ‘*XGBoost’* and ‘*SKLearn’*.

#### Best models in MAQC and SEQC project

We also compared HetEnc to the best predictive model that developed by various attendees that submitted to the consortium during the MAQC/SEQC projects. Note that these best models were not restricted to any data normalization, feature selection or modeling algorithms, and their performance was only evaluated by the testing samples, which were blinded to them when training their models. The best models were selected as using the best model of a single attendee, which included 6 microarray models and 54 RNA-seq models. The final performance of SEQC models were their average AUCs.

## Results

### Defining the HetEnc architecture

HetEnc is inspired by the domain separation network [23] developed for image analysis. The domain separation network extracts image representations into two subspaces: one private component, and another component shared by different domains. Here, we implemented a similar idea in HetEnc to represent the gene expression, to show the platform-shared (or platform-independent) information by organizing different platforms’ data into the designated encoding networks.

**Fig. 2:**
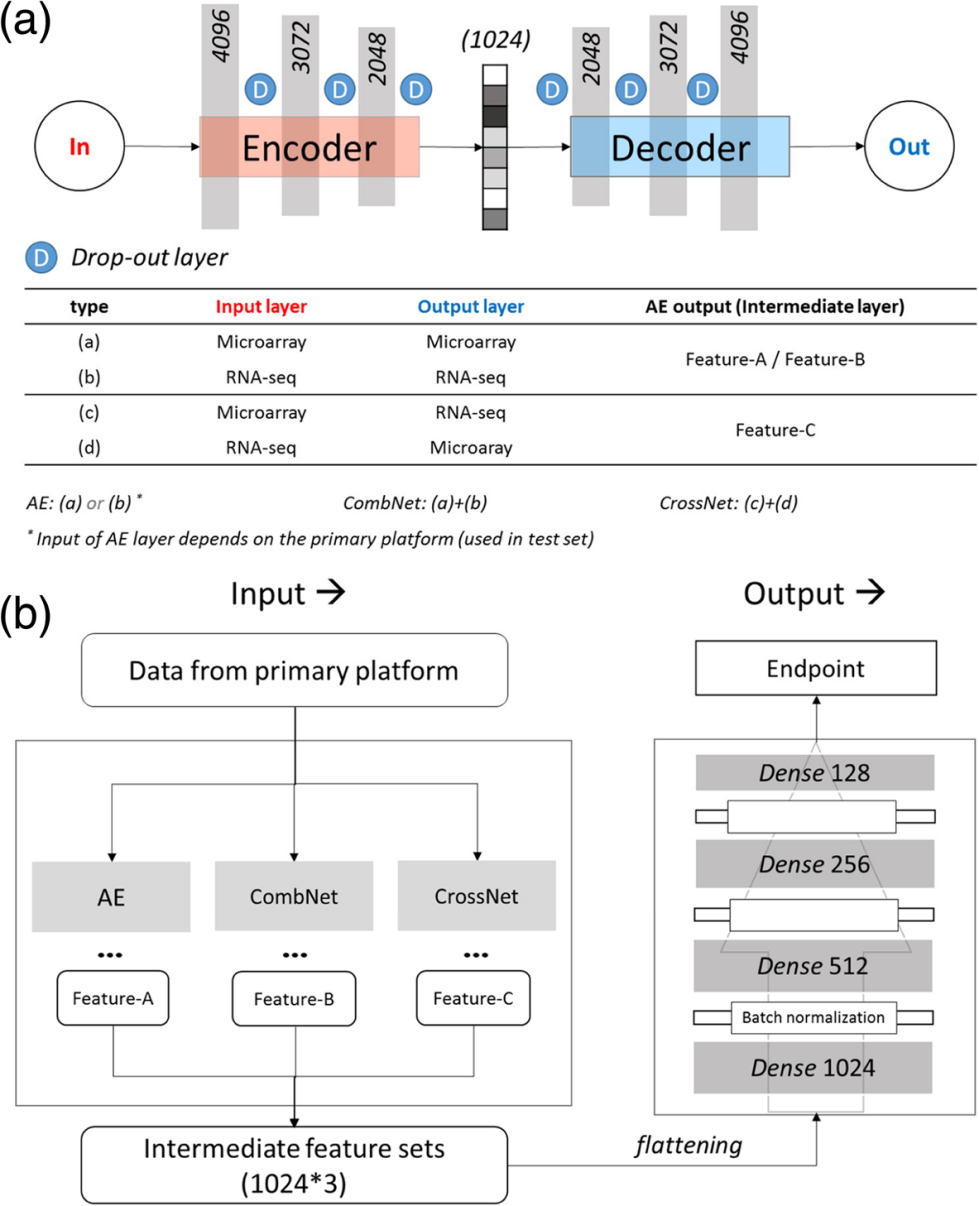
HetEnc overview. (**a**) feature representation model architecture and three different encoding networks (AE, CombNet and CrossNet) used in the study; (**b**) feature extraction and 6-DNN structure in the modeling step

The entire HetEnc architecture is composed of two modules. The first feature representation module is the key module, which is designed to extract the gene expression representation into different subspaces via different representing or encoding networks. The first module involves three distinct encoding networks; Autoencoder (AE), CombNet and CrossNet (Fig. 2a), for extracting different subspaces of the feature representation, respectively. The second module of HetEnc is the modeling step, which is basically a six-layer deep neural network (named 6-DNN) used to predict targeted endpoints using the intermediate features (Fig. 2b).

In the feature representation step, the most differences between three networks (AE, CombNet and CrossNet) are the definition of input and output data, which could be the same or different platform. In all, there are four different combinations of microarray and RNA-seq, as shown in Fig. 2a. For example, if microarray is the primary input platform, AE will use type (a). CombNet will use the input-output combination of (a) and (b). CrossNet will use the combination of (c) and (d). On the other hand, If RNA-seq is the primary input platform, AE will use type (b). CombNet will use the combination of (a) and (d), and CrossNet will use the combination of (c) and (d). Note that CombNet and CrossNet will not change when primary platform changed. The three intermediate feature sets generated by three encoding networks were named Feature-A, Feature-B and Feature-C for AE, CombNet and CrossNet, respectively.

The hyper-parameters of AE are described as follows: in total, nine layers were formed, with the number of nodes, *p*, 4096, 3072, 2048, 1024, 2048, 3072, 4096, *p*, respectively, where *p* was the number of input (and reconstructed) gene features. The first four layers are the encoding network and the last four layers are the decoding network. The compressed feature set, therefore, is from the bottleneck (i.e., intermediate) layer, with 1024 nodes. We used the hyperbolic tangent (*Tanh*) activation function for all AE hidden layers, and the sigmoid (*logistic*) activation function for the output layer. For denoising, we also added one drop-out layer between sets of two layers, and the drop-out ratio was set to 0.2.

In the modeling step, the six-layer feed-forward deep neural network (6-DNN) is depicted in Fig. 2b. The hyper-parameters of 6-DNN are listed here: (1) Network size: sizes for each layer (i.e., node) in the network are x, 1024, 512, 256, 128 and 2, respectively; where x is the size of the intermediate features set, and 2 is a categorical endpoint for a binary endpoint. For most of this study, x = 3072; when using one or two AE models in comparative analysis, the input shape would also change to 1024, 2048, respectively. (2) Activation function: we used the Rectified Linear Unit (RELU) activation function for all dense hidden layers, and Softmax activation for the output layer, as a classification task. (3) Regularization: between two hidden (dense) layers, batch normalization was added for purposes of regularization, as depicted in Fig. 2b. Due to concern over introducing bias when using batch normalization and drop-out simultaneously (Li, et al., 2018), the drop-out layer is not implemented in the 6-DNN network structure.

### Predictive performance on SEQC neuroblastoma dataset

We evaluated our model on the SEQC neuroblastoma dataset. In total, six predictive models were trained for endpoints FAV, OS_All and OS_HR, and two data platforms (Microarray, RNA-seq), respectively. Because the first step (feature representation) is unsupervised, the encoding networks (i.e., AE, CombNet, CrossNet) generated by the first step would be shared between three endpoints in the modeling step.

**Fig. 3:**
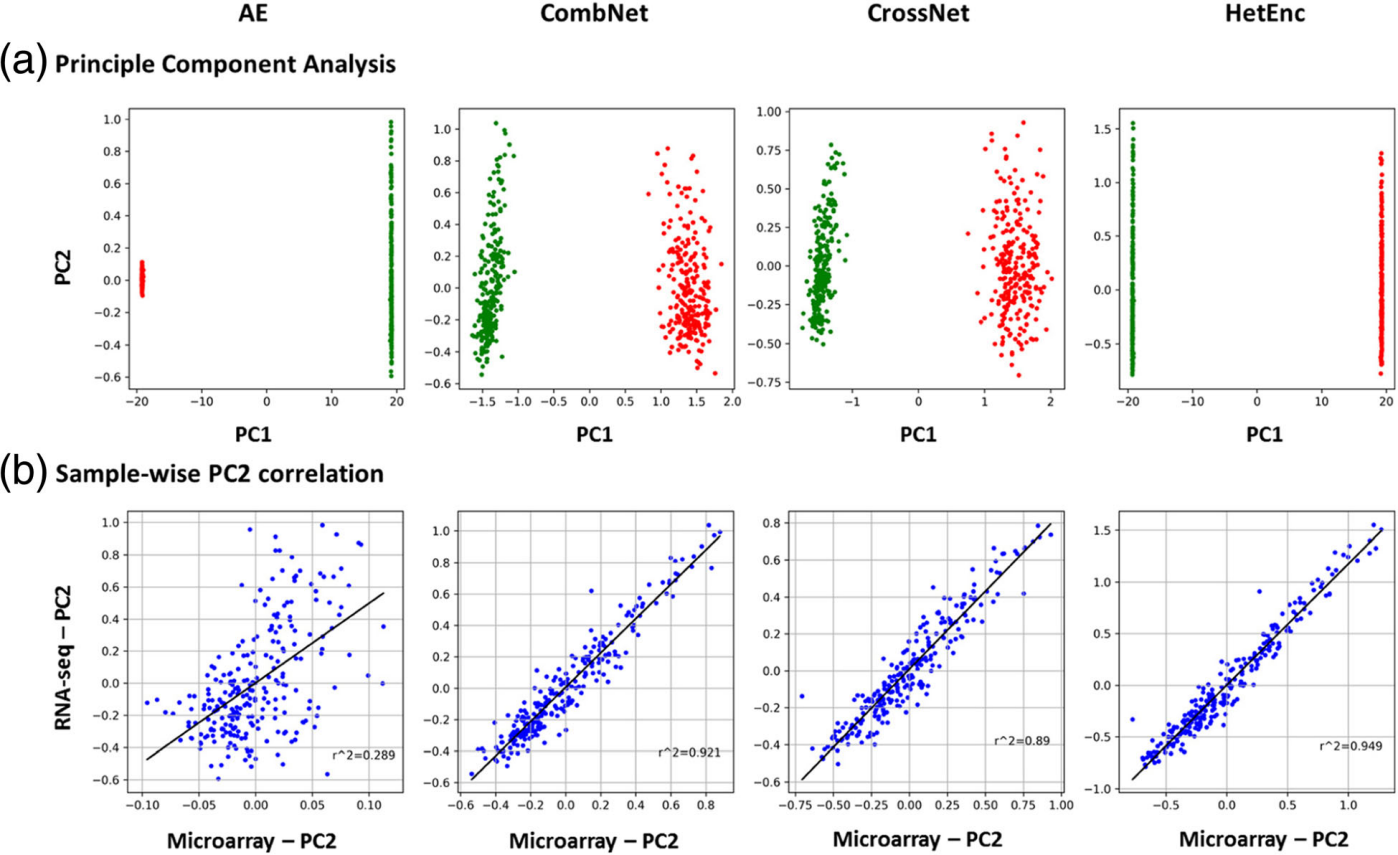
(**a**) Principle Component Analysis (PCA) by features extracted by HetEnc and its three encoding networks: AE, CombNet and CrossNet. RNA-seq and Microarray samples are combined for PCA analysis. Green and red dots represent RNA-seq and Microarray samples, respectively. (**b**) A sample-wise scatter plot of PC2 correlation analysis between Microarray and RNA-seq platform

We first applied Principle Component Analysis on the intermediate features generated by three encoding networks. Latent features from each encoding network will be analyzed both independently and combined as HetEnc features, as shown in Fig. 3a. Microarray and RNA-seq samples were combined in PCA analysis. For AE features, latent features (Feature-A) of Microarray and RNA-seq samples were generated by different AE models; For CombNet and CrossNet, the latent features (feature-B and C) were generated by the same model. As a result, we observed in all PCA results, Microarray and RNA-seq samples are highly distinguished from each other along Principle Component 1 (PC1), indicating the large inherent differences (platform-related variance) between these two platforms. Compared to AE, CombNet and CrossNet has a closer distance between Microarray and RNA-seq samples on PC1. On the other hand, Microarray and RNA-seq samples fall into a similar range of PC2 particularly in CombNet and CrossNet, implying PC2 reflected some common properties (i.e., platform-independent variance) between two platforms.

To further reveal the platform-independent variance in PC2, a correlation analysis was performed on PC2 between Microarray and RNA-seq of the same sample. As shown in Fig. 3b**,** the pair-wise correlation of PC2 from AE between Microarray and RNA-seq is not significant (r^2^ = 0.289). On the other side, there are high linear correlation of PC2 from CombNet and CrossNet between RNA-seq and Microarray, as r^2^ reached 0.921 and 0.89 respectively. Further, when combined latent features from AE, CombNet and CrossNet together as HetEnc, the linear correlation between two platforms became higher (r^2^ = 0.949). This PCA and pair-wise PC2 correlation analysis result indicated that CombNet and CrossNet could represent platform-independent features from the raw dataset.

The predictive models were first constructed and evaluated by five-fold cross-validation within the training dataset. In the five-fold cross-validation, we randomly separated the entire training dataset into five subgroups; where in each run, four subgroups of samples were used to train the model, and the remaining one was used as a testing set for evaluating the model’s performance. Each subgroup was tested once. By picking different random seeds, we repeated the five-fold cross-validation 20 times; therefore, a total of 100 (5*20) sub-models were built for each endpoint.

After evaluating the model via cross-validation, we trained one model using all 249 training samples for each endpoint. The model was finally evaluated on the test dataset with the other 249 samples. Similarly, we set different random seeds to run the whole modeling process 100 times to retrieve an average predicting performance. We measured the model’s performance for three endpoints (FAV, OS_All, OS_HR). As shown in Table 2, we observed a small standard deviation among 100 repeats in both cross-validation and external testing, indicating initial random seed had little influence on the overall performance. We also observed that the external evaluation performance was very close to the cross-validation result, confirming that our HetEnc model did not overfit the training dataset.

**Table 2:** Predictive performance (AUC) for the neuroblastoma dataset

	Model	RNA-seq			Microarray		
		FAV	OS_All	OS_HR	FAV	OS_All	OS_HR
Cross-validation	HetEnc	0.964 (0.009)	0.830 (0.019)	0.520 (0.044)	0.962 (0.011)	0.849 (0.024)	0.651 (0.044)
	HetEnc	**0.969** **(0.007)**	**0.854** **(0.024)**	**0.592** **(0.027)**	**0.948** **(0.015)**	**0.825** **(0.016)**	**0.569** **(0.022)**
	Raw-DNN^*^	0.926 (0.043)	0.698 (0.058)	0.578 (0.03)	0.906 (0.054)	0.721 (0.035)	0.568 (0.031)
	FS-DNN^*^	0.923 (0.052)	0.704 (0.046)	0.558 (0.028)	0.919 (0.056)	0.722 (0.047)	0.559 (0.025)
External Testing (on same testing set)	KNN	0.896 (0.032)	0.641 (0.032)	0.495 (0.048)	0.907 (0.035)	0.662 (0.031)	0.515 (0.041)
	NSC	0.901 (0.036)	0.700 (0.048)	0.499 (0.036)	0.921 (0.032)	0.713 (0.067)	0.510 (0.035)
	SVM	0.894 (0.043)	0.631 (0.024)	0.512 (0.050)	0.914 (0.035)	0.620 (0.034)	0.525 (0.047)
	RandomForest	0.905 (0.014)	0.740 (0.019)	0.563 (0.030)	0.912 (0.012)	0.727 (0.020)	0.560 (0.030)
	XGBoost	0.883	0.742	0.517	0.874	0.749	0.611
	Avg. of Best 60 SEQC Models	0.931 (0.02)	0.735 (0.072)	0.544 (0.052)	0.929 (0.02)	0.756 (0.082)	0.563 (0.038)

^*^Raw-DNN used the raw 10,042 gene features as input of DNN model, FS-DNN further applied feature selection threshold (*p* < 0.05 for each endpoint) before entering the DNN model. The structure of DNN model used in Raw-DNN and FS-DNN are the same as the DNN used in HetEnc supervised learning step

We compared our model performance to three machine learning models - support vector machine (SVM), nearest shrunken centroids (NSC) and k-nearest neighbors (KNN) -using the same training/testing sample distribution and data preprocessing (i.e., using the same 10,042 genes). A detailed modeling process of SVM, NSC and KNN can be found elsewhere [24]. Furthermore, we compared our result to the submitted best models from all analysis teams in the SEQC/MAQC consortium [4], which held the same data distribution (i.e., training/testing split), but had no restrictions for machine learning methods (i.e, data normalization, feature selection, modeling algorithm, etc.) or expression datasets. The SEQC predictive models were built during the SEQC project, where a total of 6 microarray and 54 RNA-seq models were constructed, and we used Area under the Receiver Operating Characteristic Curves (AUC) for comparison.

The performances (AUC) of HetEnc, KNN, NSC, SVM and the best model from SEQC/MAQC for three Endpoints (FAV, OS_All and OS_HR) with two platforms (RNA-seq and Microarray) was shown in Table 2. Since OS_HR in average showed a low performance regardless of the platform and modeling algorithm, we notated this endpoint is not predictable by current dataset and its performance would not affect the comparison result. After all, we observed that these best models from SEQC analysis teams showed better overall performance than the models constructed by KNN, NSC and SVM in the previous study. One possible explanation could be the restriction of genes to those with one-to-one mapping between RNA-seq and microarray. However, this restriction did not have any detrimental effect for our HetEnc model. As a result, our HetEnc model still showed a significantly better predicting performance (*p* < 0.01) than the best fine-tuned predictive models from the SEQC community.

## Discussion and conclusion

By developing HetEnc, the underlying hypothesis is that we assume the gene expression profiling value is determined by two factors: platform-independent factor and platform-related factor; where the platform-independent factor is mostly attributed to the sample itself, and the platform-related factor is specific to the platform used to measure the expression value. Thus, the main goal of HetEnc is to separate the information from these two factors, to reduce the noise (component) introduced by the platform and emphasize the platform-independent factor.

As demonstrated in this study, HetEnc outperformed previously-reported machine learning models overall, achieving a significantly better predictive performance. Three aspects accounted for its performance: (1) By using exact the same dataset (i.e., the same pre-processed data as model input), HetEnc showed significantly better predicting performance than such machine-learning algorithms as support vector machine (SVM), nearest shrunken centroids (NSC) and k-nearest neighbors (KNN). We observed this superior performance from both “head-to-head” comparative analysis and previously-published results. (2) With no restrictions on data pre-processing and modeling strategies, HetEnc still performed better than the best models developed by other groups in the SEQC project. (3) Performance differences between cross-validation and external testing are relatively small in developed HetEnc models, indicating that the HetEnc model can be applied more generally to new test sets.

For high-dimensionality biological data analysis, feature selection and feature representation are two most applied strategies for dimension reduction purpose. Feature selection methods aim to extract a subset of meaningful biological entities (i.e., biomarkers) from the raw data. Common feature selection methods are based on the feature itself (e.g., feature removal with low variance), its coefficients with other features (e.g., LASSO), or coefficient with the endpoint (e.g., T-test, Fold-Change, etc.). One advantage of using feature selection is about the model interpretability, as discovered biomarkers like genes or proteins could be intuitively interpreted with biological functions. However, if the relationship between the biomarkers and endpoint could be simply interpreted (such as gender-related genes and gender prediction), usually a linear/logistic regression model will be good enough to capture the relationship. On the contrast, many studies have demonstrated that the advanced modeling algorithms such as Random Forest and SVM could significantly improve the predictive performance especially for “moderate and difficult to predict” biological endpoints, indicating the relationships between biomarkers and the endpoint could be too complicated to be interpreted in a simple format. That is why we developed a feature representation method as HetEnc to learn latent features of raw inputs from multi-platforms via deep encoding networks. Still, for “easy to predict” endpoint, we believe using simple model such as KNN might be OK due to its higher biological relevance and interpretability, without losing too much performance. On the other hand, for “unpredictable” endpoints such as OS_HR in this study, any modeling algorithm would not have significant differences.

Moreover, unlike other integrated modeling which combined features from different platforms either horizontally or hierarchically [7], one unique advantage of HetEnc is that it does not require multi-platform data for the test samples. HetEnc is designed to use multi-platform data to train two hetero-encoding networks in the feature representation step, which is totally unsupervised; therefore, no labeling information (i.e., the endpoint) is needed for utilizing the multi-platform data. After the encoding networks are built, HetEnc converts the input platform data into intermediate features with regards to the multi-platform information it has learned.

Additionally, these two steps are quite independent of each other, which enables HetEnc to maximize its data utilization capability. For example, many datasets have unlabeled samples; although these samples cannot be used in supervised learning (i.e., the modeling step), they could still be used in the feature representation step to further fine-tune the model parameters. Further, the modulization of HetEnc also enables the new deep learning technologies and data format to be easily embedded. For example, HetEnc could also be applied to data with spatial information, such as changing the basis architecture in the feature representation step from autoencoder to convolutional autoencoder, recurrent autoencoder, etc.

In current study, we developed the HetEnc architecture by combining AE, CombNet and Cross Net. To determine the synergic effect among these three encoding networks, we evaluated the model based on only one single encoding network. As a cross-validation result on OS_All endpoint, models with only AE, CombNet or CrossNet yield 0.808, 0.801 and 0.811 in AUC respectively, where the model with a combination of three performed better (0.830 in cross-validation and 0.854 in testing, Table 2). As many other types of encoding networks are still under explore, further optimizing the encoding networks in HetEnc would be a challenging but rewarding work to better handle multi-omics data.

Model interpretability is one of the limiting factors of many deep learning models. For example, in word embedding study, although the exact value of one word-vector have no meaning however its relative value (i.e., the distance between two word-vectors) could reflect the similarity between two words. Similarly, in HetEnc the real value of latent features (generated by encoding networks) may not have explicit biological meaning however the distance of latent features between two samples may reflect their similarity/closeness in phenotype. In this study, we applied Principle Component Analysis (PCA) to visualize these latent features from three different encoding networks as their 2D projection. As the result shown in Fig. 3, the two major components, PC1 and PC2 showed distinguished properties, as PC1 reflected more platform-related features where PC2 represented more platform-independent features. Particularly, we found the PC2 between Microarray and RNA-seq of the same sample correlated quite well in CombNet and CrossNet, indicating these two encoding networks contributed more on platform-independent features.

As we are entering the big data era, more and more researches would include multi-type data that generated for a consensus decision making, where HetEnc is one promising way to bridge different types of data by identifying their common and specific features. In this study, we restricted HetEncs to multi-platform gene expression data analysis due to the resource we now have in hand. However, the concept of HetEnc may not only suitable for the platform-specific information, but also apply to find a common information between different sites/labs, different time/dose, or different organ/tissues. Further experiments are needed to see whether the concept of HetEnc may also apply to other multi-type data researches.

In this study, we only evaluated HetEnc on a two-platform dataset, and we did not discuss multi-platform (> = 3) data analysis. Multi-platform data analysis would be more complicated, due to data sparsity and feature mapping issues, especially finding/developing a mapping system among all platforms would be difficult. Nevertheless, HetEnc holds the potential to expand to multi-platforms. A possible way for HetEnc to work with multi-platform datasets is to design more comprehensive CombNet and CrossNet models. For example, the CombNet could combine multiple platforms, and the CrossNet could represent the two-way translation between every two platforms (such as A-B; B-A; A-C; C-A; B-C; C-B for three platforms). Another strategy is to design multiple CombNet and CrossNet models. There may be one specific CombNet and CrossNet model for each platform pair, thus combining the features from all representation models, this strategy may allow to avoid developing CombNet and CrossNet models for two rarely related platforms due to lack of mapping system. Still, further investigation and optimization is essentially needed to generalize HetEnc to multi-platform data analysis, as well as to sparse datasets for which including samples that not have multi-platform data.

## Data Availability

The source code of HetEnc is available at Github (https://github.com/seldas/HetEnc_Code). SEQC Neuroblastoma dataset is publicly available at GEO (GSE49710 and GSE62564).
